# EaveTubes for control of vector-borne diseases in Côte d’Ivoire: study protocol for a cluster randomized controlled trial

**DOI:** 10.1186/s13063-023-07639-9

**Published:** 2023-11-02

**Authors:** Raphael N’Guessan, Serge-Brice Assi, Alphonsine Koffi, Phamien Ludovic Ahoua Alou, Anatole Mian, Nicole L. Achee, Benedicte Fustec, John P. Grieco, Fang Liu, Santosh Kumar, Matthew Noffsinger, Ashley Hudson, Tim W. R. Möhlmann, Marit Farenhorst

**Affiliations:** 1 Vector Control Product Evaluation Centre/Institut Pierre Richet, Bouaké, Côte d’Ivoire; 2 University of Notre Dame, Notre Dame, IN USA; 3 In2Care BV, Wageningen, Netherlands; 4 Keough School of Global Affairs, University of Notre Dame, Notre Dame, IN USA

**Keywords:** EaveTubes, Housing, Malaria control, LLINs, Cost-effectiveness, Malaria, Vector-borne diseases, Mosquito vectors, Incidence

## Abstract

**Background:**

Vector control tools, long-lasting insecticidal nets (LLINs) and indoor residual spraying (IRS), have significantly contributed to malaria prevention efforts in sub-Saharan Africa. However, insecticide resistance has seriously hampered their efficacy in recent years and new tools are essential to further progress. In2Care® EaveTubes (ETs) are an inexpensive, new resistance-breaking vector control product under World Health Organization (WHO) evaluation informed by mosquito ecology to efficiently target malaria vectors. By installing ETs in the walls of the house at the eave level that funnel the natural airflow, mosquitoes are drawn in by the same heat and odor cues that typically attract them through open eaves. Once inside an ET, mosquitoes are exposed to insecticide-treated netting placed inside the ET. The aim of this study is to test whether ETs as stand-alone tool have an effect on the epidemiology of malaria in villages where houses have been modified with the ET intervention.

**Methods:**

A two-armed, cluster randomized controlled trial will be conducted to evaluate the effect of ETs on clinical malaria incidence in children living in Côte d’Ivoire. Thirty-four villages will be selected based on population size and the proportion of houses suitable for modification with ETs (17 treatment arms (ETs + LLINs, 17 control arms (LLINs only)). Based on the population census, 55 households per cluster with eligible children (i.e., between the ages of 6 months to 8 years old at the start of the study) will be randomly selected for recruitment into the active detection cohorts. In the treatment arm, we will enroll eligible children who reside in ET-treated houses. The intervention and control cohorts will be followed for 4 months for baseline covariate measurements and 24 months with intervention. During case detection visits, blood samples will be taken from all febrile children and tested for malaria infection with rapid diagnostic tests (RDTs). All positive clinical malaria infections will be treated. To estimate the impact of the ET on malaria vector densities, entomological measurements (indoor sampling with CDC traps) will be conducted monthly in 20 clusters (10 ET, 10 Control) in 10 randomly selected households per cluster. To estimate the infectiousness of malaria vectors, sporozoite rates will be measured in subsets of the collected mosquito samples.

**Discussion:**

Findings will serve as an efficacy trial of ETs and will be submitted to the WHO Vector Control Advisory Group (VCAG) for assessment of public health value. Entomological outcomes will also be measured as proxies of malaria transmission to help develop guidelines for the evaluation of future In2Care® ETs products.

**Trial registration:**

ClinicalTrials.gov NCT05736679. Registered on 10 February 2023.

**Supplementary Information:**

The online version contains supplementary material available at 10.1186/s13063-023-07639-9.

## Administrative information

Note: the numbers in curly brackets in this protocol refer to SPIRIT checklist item numbers (Additional file 1). The order of the items has been modified to group similar items (see http://www.equator-network.org/reporting-guidelines/spirit-2013-statement-defining-standard-protocol-items-for-clinical-trials/).

**Table:** 

Title {1}	EaveTubes for Control of Vector Borne Diseases in Côte d’Ivoire
Trial registration {2a and 2b}.	ClinicalTrials.gov Identifier: NCT05736679. Registered February 10, 2023. https://clinicaltrials.gov/ct2/show/NCT05736679
Protocol version {3}	July 17, 2022 – Version 4.0
Funding {4}	This trial has received financial support from the Fund for Innovation in Development (Grant no. AFD CCII9O8 OIM), USAID Development Innovation Ventures (Grant no. 7200AA21FA00042), and the Achmea Foundation (Agreement 19 Dec 2022).
Author details {5a}	Raphael N’Guessan, Vector Control Product Evaluation Centre/Institut Pierre Richet, Côte d’Ivoire Serge-Brice Assi, Vector Control Product Evaluation Centre/Institut Pierre Richet, Côte d’Ivoire Alphonsine Koffi, Vector Control Product Evaluation Centre/Institut Pierre Richet, Côte d’Ivoire Phamien Ludovic Ahoua Alou, Vector Control Product Evaluation Centre/Institut Pierre Richet, Côte d’Ivoire Anatole Mian, Vector Control Product Evaluation Centre/Institut Pierre Richet, Côte d’Ivoire Nicole L. Achee, University of Notre Dame, USA Benedicte Fustec, University of Notre Dame, USA John P. Grieco, University of Notre Dame, USA Fang Liu, University of Notre Dame, USA Santosh Kumar, University of Notre Dame, USA Matthew Noffsinger, University of Notre Dame, USA Ashley Hudson, University of Notre Dame, USA Tim W.R. Möhlmann, In2Care BV, Netherlands Marit Farenhorst, In2Care BV, Netherlands
Name and contact information for the trial sponsor {5b}	In2Care BV Marijkeweg 22, 6709 PG Wageningen, The Netherlands Email: marit@in2care.org Tel: + 31 (0)317–769,018
Role of sponsor {5c}	Medical entomologists from In2Care BV gave advice on the study design and contributed to the protocol development, but will hold no authority over the study design, collection, management, analysis/ interpretation of data, writing of the report, or the decision to submit the report for publication. The trial funders had no role in and will hold no authority over the study design, collection, management, analysis/ interpretation of data, writing of the report, or the decision to submit the report for publication. Our grant agreements include a clause that the beneficiary remains free to use and/or publish the outcomes or results of the trial in public reports, publications or external communications.

## Introduction

### Background and rationale {6a}

Control efforts in sub-Saharan Africa over the past 15 years have prevented an estimated 663 million clinical cases of malaria caused by *Plasmodium falciparum* [1]. Vector control, either in the form of long-lasting insecticidal nets (LLINs) or indoor residual spraying (IRS), is estimated to be responsible for 78% of those averted cases [2]. However, insecticide resistance has seriously hampered their efficacy in recent years.

Because of the impact of vector control on malaria prevalence, it is essential that existing tools are preserved and that cost-effective, environmentally friendly, and socially acceptable new tools are developed. The benefit of having new tools is two-fold: there is the ability to control mosquitoes that are not being controlled by existing interventions (e.g., insecticide-resistant mosquitoes or outdoor biting mosquitoes), and new tools provide options for managing insecticide resistance in mosquitoes, for example through “combination therapy” with two or more insecticides, analogous to using multiple drugs to combat drug resistance in parasites [3].

The local malaria vector populations in Côte d’Ivoire are highly resistant to almost all classes of insecticides used for vector control, and studies showed a high resistance ratio of local *Anopheles gambiae* relative to susceptible vectors when exposed to deltamethrin [4]. One recent study in the trial area showed high levels of insecticide resistance against pyrethroids in *Anopheles gambiae s.s*. and *Anopheles coluzzii* vectors. Across study villages, dose–response assays demonstrated the resistance intensity to deltamethrin was extremely high (> 1500-fold), and mortality following exposure to pyrethroid-treated bed nets was low (< 30% mortality in cone bioassays) [5].

In2Care® EaveTubes (ETs) could help meet the pressing need for new vector control tools. ETs are an inexpensive, field-ready technology, informed by mosquito ecology to efficiently target malaria vectors. ETs are a home improvement that, in addition to the physical blocking, provides a mosquito-killing effect that should lead to a community-level impact on malaria when implemented at scale. Mosquitoes that transmit malaria in sub-Saharan Africa utilize odor cues to enter homes and search for hosts to blood feed. Anophelines were shown to have a strong preference for entering traditional-style African homes through gaps between walls and the roof—i.e., the eaves of houses [6–9]. Closing off the eaves of such traditional-style houses with netting or curtains was shown to provide a physical barrier that can selectively prevent malaria mosquito entry into the house and, consequently, protect inhabitants from malaria [10–12]. In this case, it is the physical blocking of mosquito entry into the house that is the major benefit of house improvements in controlling malaria [12], but that does not assert a vector-killing impact and could lead to deflection to untreated households. Moreover, modern-style housing in Africa is showing a strong tendency towards closed eaves. ETs comprise 6-in. tubes that can be installed at eave level in traditional and modern houses, which funnel the natural airflow and lure mosquitoes in by the same heat and odor cues that typically attract them through open eaves. Once inside an ET, mosquitoes are exposed to insecticide-treated netting placed inside the ET.

In2Care® ETs have been designed with netting inserts that are covered in a coating with an electrostatic charge to hold powder formulations of insecticides. Mosquitoes contacting the static netting pick up a large dose of insecticide, overcoming insecticide resistance in the mosquito. Studies showed that pyrethroid-resistant anophelines could be effectively killed with pyrethroid-treated static netting [13] and that pyrethroid-treated ETs have high efficacy on vector and malaria incidence in an area of intense insecticide resistance [14]. Since the netting inserts are small and placed in locations that are not contacted commonly by residents (at roof level), the quantity and risk of exposure to insecticide is small. It should, therefore, be possible to use ETs as a delivery system and develop future product versions with a wide variety of insecticides that can, in future, be rotated or combined to prevent resistance as recommended by WHO.

Semi-field studies in Tanzania, Kenya, and Côte d’Ivoire demonstrated attraction of mosquitoes to the ETs and a reduction in overnight survival [15–17]. In a cluster randomized control trial conducted in Côte d’Ivoire between 2016 and 2019, ETs + window screenings were shown to reduce malaria case incidence by 38% [14] and by 47% in villages with > 70% coverage. The intervention provided community protection (27% less malaria in untreated houses) and significant reductions in anemia and mosquito densities. The World Health Organization Vector Control Advisory Group (WHO VCAG) confirmed the results of the trial in Côte d’Ivoire contribute substantially to the evidence base on ETs and window screening, recommended epidemiological trials with the stand-alone product, and approved the study protocol for this new trial in Côte d’Ivoire [18].

ETs were originally tested in combination with untreated window screening (“SET”), in order to block other potential entry points and funnel mosquitoes towards the ET. While window screening alone could protect individuals at the household level, it is likely to have less impact on community levels of malaria. Semi-field studies done with ETs without window screens suggest that the large impact observed in the first trial may have been primarily afforded by the ETs [19]. Window screening is difficult to standardize per house and window type, is costly, and needs frequent repairs, and is not easy to scale. Nevertheless, economic evaluations showed that SET was already similarly cost-effective to other currently used tools like IRS. However, if the intervention consisted only of ET and good epidemiological impact was still demonstrated, the cost-effectiveness would be substantially greater.

The aim of this study is to test whether ETs as stand-alone tool have an effect, not only on mosquito populations but also on the epidemiology of malaria in villages where houses have been modified with the ETs. Another trial is already ongoing in the Jinja area of Uganda: a 3-arm RCT evaluating malaria impacts of ETs *versus* full house screening (windows/door/gaps) and versus routine vector control (PBO bed nets). This USAID/CDC-funded “Uganda Housing Modification Study” (https://clinicaltrials.gov/ct2/show/NCT04622241) installed ETs in 2000 households in 20 clusters and is monitoring the incidence of clinical malaria through passive case detection at local Health Centers during 1 year. Together, these 2 clinical trials with ETs as stand-alone tools will be submitted to the WHO VCAG to provide the evidence base for the public health impact of In2Care® ETs.

The study is being conducted in the same region of Côte d’Ivoire as the first SET trial, where insecticide resistance is widespread in the mosquito population. This is necessary to demonstrate that (pyrethroid-treated) ETs could potentially provide additional protection, even in areas where existing tools (i.e., LLINs) may be compromised by insecticide resistance. Our study is designed to test any added benefit of the ET package on top of standard control measures. By executing this trial in the same region with similar design, results of the ET intervention will be comparable with the previously achieved SET impact results.

## Objectives {7}

### Main research question

This study intends to test whether ETs protect people against mosquitoes (entomological endpoints) and malaria (epidemiological endpoints) in an area where malaria transmission is driven by insecticide-resistant *Anopheles gambiae* mosquitoes.

The primary study objective is to assess whether ETs reduce the number of malaria infections and clinical malaria, defined by active case detection, in children between 6 months and 10 years of age, compared to children living in communities without ETs, in an area where there is universal coverage of LLINs (1 LLIN for every 2 people) and pyrethroid resistance is high.

Secondary objectives include:

(i) To determine how ETs impact the rate of anemia (moderate anemia defined as 7–9.9 g/dL hemoglobin; severe anemia as < 7 g/dL hemoglobin)
(ii) To assess how ETs impact Entomological Inoculation Rates (EIR) computed as the product of the anopheline vector density and the sporozoite rate, compared with LLINs alone
(iii) To assess the cost-effectiveness of the ETs intervention
(iv) To assess the user acceptance of the ETs intervention

## Trial design {8}

The trial design is a 2-armed cluster randomized control trial (cRCT) with 17 clusters (villages) per arm for evaluating the protective efficacy of the SR intervention. Clusters are the unit of replication in this design, and they will be randomly allocated to one of the two arms. Both the replication and randomization in our design are essential to distinguish the effect of ETs from other village-level variation in malaria incidence.

Villages that have not participated in the previous SET cRCT, and villages that received standard pyrethroid-only LLINs (Permanet 2.0) from the National Malaria Control Program (NMCP) distribution campaign in May 2021 will be enrolled. We will exclude villages being treated by IRS and/or new generation bed net campaigns.

The control arm of this cRCT will include 17 villages with universal coverage of LLINs (defined as 1 LLIN for every 2 people) and no ETs. The 17 villages in the treatment arm will receive universal coverage of LLINs plus ETs. For those households assessed to not have met thresholds of universal coverage, topping up of LLINs will be conducted as needed.

## Methods: participants, interventions, and outcomes

### Study setting {9}

The study is being conducted in the Béoumi district in the Gbêkê region in central Côte d’Ivoire where there is year-round malaria transmission with a peak during the wet season (May–October). This is the same region of Côte d’Ivoire as the first trial, where on average malaria incidence was 2–3 malaria cases/child/year and insecticide resistance is widespread in the mosquito population. This is necessary to demonstrate that (pyrethroid-treated) ETs could potentially provide additional protection, even in areas where existing tools (i.e., LLINs) may be compromised by insecticide resistance. Our study is designed to test any added benefit of the ET package on top of standard control measures. By executing this trial in the same region with similar design, results of the ET intervention will be comparable with the previously achieved SET impact results.

### Eligibility criteria {10}

Village-level inclusion criteria:

-  ≥ 80% of households (HHs) must be suitable for ET installation.
-  ≥ 70% of HHs willing to have ETs installed.
- No participation in the previous screening + ETs cRCT.
- Received standard pyrethroid-only LLINs (Permanet 2.0).
- 100–300 HHs per village.
-  ≥ 2 km away from another village.

Village-level exclusion criteria:

-  < 80% of HHs suitable for ET installation
-  < 70% of HHs willing to have ETs installed
- Villages being treated by IRS and/or new generation bed net campaigns
- Participation in previous Screening + ET cRCT
-  < 100 and > 300 households per village
-  < 2 km from another village

Household-level inclusion criteria:

- HHs must be suitable for ET installation
- Provision of consent from heads of HH

Household-level exclusion criteria:

- HH not suitable for ET installation (e.g., houses with poor quality thatch roofing or very large eaves or wall gaps, houses in substantial disrepair, unfinished houses under construction, poorly constructed houses)
- No provision of consent from heads of HH

Individual-level inclusion criteria:

- Children aged ≥ 6 months to < 8 years old at the time of enrollment (so all participants are under 10 years old for the duration of clinical follow-up).
- Provision of written, informed consent by parents/caregivers.
- Children must reside in villages enrolled in the study and in ETs-treated HHs.
- Hemoglobin at baseline of > 7 mg/dL.

Individual-level exclusion criteria:

- Children aged < 6 months or ≥ 8 years old at the time of enrollment
- No provision of written, informed consent by parents/caregivers for child participation
- Expected to be non-resident during a significant part of the transmission season
- Hemoglobin at baseline of ≤ 7 mg/dL, have a known chronic disease, or have signs of clinical decompensation
- Participation in another clinical trial investigating a drug, vaccine, medical device, or procedure

### Who will take informed consent? {26a}

The study subjects may understand French, and/or Baoulé, and/or Malinke. The consent forms will be translated from English into French and then from French into Baoulé, or Malinke by members of the team. These two local languages are only oral; they are not written. The translated version will be on a vocal recording. The consent team of trained, study personnel will be trained to provide the same translation of the survey in Baoulé and Malinke.

The consent process for participation in the active infection detection cohort will occur at the subjects’ home. After the recruitment material and screening questions have been presented to the parents, if the child meets the screening criteria, a team member will show the consent form to the parent. The team member presenting the consent form will be fluent in both French and Baoulé or Malinke. If the parent feels uncomfortable reading the form in French, the team member will help the parent by reading the form to the parent and/or providing an oral translation of the form in Baoulé or Malinke. The local (Ivorian) researchers do not typically provide compensation for participating in epidemiological studies. We will follow these customs and not provide compensation for participating.

The consent process for ETs’ installation in households will take place at the residents’ homes in the 17 selected intervention villages. Once the house is deemed eligible for ETs’ installation, a team member will present the consent form to the head of household. The team member presenting the consent form will be fluent in both French and Baoulé or Malinke. If the head of household feels uncomfortable reading the form in French, the team member will help by providing an oral translation of the form in Baoulé or Malinke.

The consent process for entomological monitoring in households will take place at the participants’ homes. randomly selected each month in the same way as described above for the ETs’ installation consents.

The informed consent form will be provided on paper. The team member will read the recruitment information and the screening questions in both French and Baoulé or Malinke. If the participant meets the screening criteria, the consent form will be read and at the bottom of the page, the participants will be asked to sign the form, which will serve as their agreement to participate. A witness will also be asked to sign the form.

Participants are free to withdraw from the study at any time and they will be informed of this right during the informed consent process.

### Additional consent provisions for collection and use of participant data and biological specimens {26b}

Not applicable. No ancillary studies will be conducted with participant data.

### Interventions

#### Explanation for the choice of comparators {6b}

According to the WHO VCAG’s guidelines for vector control field trial design, studies should always have a control arm from which data is collected simultaneously with data collection from an intervention arm [17]. The control arm of this cRCT will include villages with universal coverage of pyrethroid-treated LLINs and no ETs.

The cRCT study design will not withhold standard-of-care for clinical management of malaria. Study participants will also not be instructed to avoid alternative vector control tools (e.g., coils, topicals, insecticide-treated nets).

#### Intervention description {11a}

In2Care® ETs comprise 15-cm diameter, 10–20 cm long ventilation tubes with removable netting inserts that are placed in the wall at eave level under the roof of houses where they attract malaria mosquitoes at night, block them from entering the house, and contaminate them with a lethal dose of insecticide. In2Care® ET netting inserts have an electrostatically charged coating treated with bio-actives in powder form, which kills insecticide-resistant mosquitoes through high active ingredient (AI) dose transfer. ETs represent a novel way of delivering an insecticidal AI. In principle, any AI that kills a mosquito or otherwise reduces its ability to transmit malaria parasites could be used, subject to satisfying the appropriate safety requirements and operational factors such as persistence. As such, the specific nature of the AI is not central to the technology or the cRCT.

For the purpose of the trial, we will use a commercially available 5% dustable powder formulation of the pyrethroid insecticide deltamethrin (K-Othrine 50WP, Bayer). This formulation was shown to be effective and persistent against resistant anophelines in Côte d’Ivoire and is used in ET product registrations.

ETs’ installations will be carried out by trained local builders under the day-to-day supervision of the local Principal Investigator (PI) and senior field staff. The aim is to achieve on average a > 70% ET coverage in the intervention clusters. Eligible households in the intervention clusters will receive on average 8–10 ETs per house. The ET intervention consists of five elements:

1. House modification: In houses with concrete/hard brick walls and closed eaves, we will drill 16-cm diameter holes (to fit the tubes) approximately 20 cm below the roof at 1.5–2-m intervals into the outer walls of rooms occupied in evening/at night (bedrooms and living rooms but not storage rooms). Per room, 2 ETs will be placed at minimum in opposite walls to maximize airflow. Where possible, ETs will be fitted in eave openings or behind air vents to avoid the need for drilling. The remaining open eave spaces or gaps in the walls will be sealed with brick, cement, or plaster. Houses with large open eaves (> 40 cm) will not be eligible and are excluded.
2. ETs’ installation: PVC tubes of 10–20-cm length and 15-cm diameter will be installed behind air vents, in eave gaps, or in the drilled holes and fixated with cement. These installed tubes will be fitted with untreated ET netting inserts immediately after placement to avoid mosquito entry.
3. Insecticide treatment: After all cluster ET installations are complete, untreated inserts will be replaced with insecticide-treated netting inserts. This will be done within a 2-week timeframe to ensure a simultaneous start in all intervention clusters. Deltamethrin powder will be applied locally with custom-built closed-system applicators, similar to the first SET cRCT.
4. Maintenance: The condition of the ET intervention will be monitored through village “walk-throughs” every 4 months. Any damage to ETs or walls will be recorded and repaired.
5. Insecticide retreatment. A random sample of 15 inserts in total will be collected from 5 randomly selected HHs in ET clusters to monitor the persistence of the insecticide on a bi-monthly basis using bioassays with local, field-collected (pyrethroid-resistant) mosquitoes. All inserts in the intervention households will be replaced with freshly treated inserts if bioassay mortality falls below 70%. Retrieved used ET inserts will be washed, dried, and retreated for the next servicing round.

#### Criteria for discontinuing or modifying allocated interventions {11b}

A study participant will be discontinued from participation in the study if:

- Withdrawal of consent by subject or parent of cohort subject.
- Cohort subject is non-resident for a significant portion of the malaria transmission season.
- Cohort subject is not available for follow-up visits (i.e., lost to follow-up).
- Subject experiences any clinically significant adverse events (AEs), laboratory abnormalities, or other medical conditions or situations such that continued participation in the study would not be in the best interest of the subject. This includes events that are not related to malaria or the ET intervention.
- Development of any exclusion criteria.

The reason for participant’s premature termination will be documented on the appropriate page of the data collection forms and specified which of the following possible reasons were responsible for the study’s premature termination:

- Serious adverse event (SAE); any events that are life-threatening or result in death, events that result in hospitalization or prolongation of existing hospitalization, events that result in persistent or significant debilitation or incapacity.
- Participant’s consent withdrawal.
- Lost to follow-up: A “lost to follow-up” is any participant who completed all protocol-specific procedures up to the administration of the investigational product or intervention, but was then lost during the study period to any further follow-up, with no safety information and no endpoint data.
- Any other reason requiring a premature termination of the participant.

All study participants are free to withdraw from the study at any time without giving a reason. If a subject voluntarily withdraws or is withdrawn by the Principal Investigator (PI) during the study, data from their follow-up until that date will be used towards incidence analyses. A 20% LTFU was included in the sample size calculations, so there will be no new children recruited as replacement for follow-up during the study.

#### Strategies to improve adherence to interventions {11c}

The status of the intervention (damage to ETs) and general condition of all houses will be monitored by quarterly village walk-throughs by project staff in both study arms. In addition, a designated member of the study team will be available for householders to report ET-related construction problems and get them fixed during the trial.

The persistence of the chemical insecticide used on the inserts will be monitored bimonthly by taking a sample of inserts from the treated villages to the lab (these will be replaced with fresh inserts) and exposing mosquitoes to them in a controlled bioassay. We will use F1 adult female anopheline mosquitoes reared from field-collected eggs in WHO cone tests with a 3-min exposure and mortality monitored 1 day post-exposure. Mortality will be compared against equivalent mosquitoes (wild-type resistant) exposed to untreated “control” inserts. Inserts will be replaced once the mortality post-exposure falls below 70% (from previous results, we expect this to be every 10–12 months).

During ET insert retreatment rounds, the number and quality of the retrieved inserts will be monitored as an indicator of product quality and intervention adherence.

#### Relevant concomitant care permitted or prohibited during the trial {11d}

While the standard-of-care for clinical management of malaria and vector control interventions (e.g., LLINs, IRS) will not be withheld in either the study arm, these interventions will be monitored and recorded throughout the trial. At baseline, children enrolled into the cohorts will be provided a 3-day course of standard, first-line antimalarials (Coartem® or ASAQ Winthrop®, both Artemisinin combination therapies (ACTs) recommended by the NMCP in Côte d’Ivoire) to clear any malaria parasite infections as well as a new LLIN. In addition, subjects will be provided treatment for malaria infection throughout the follow-up period. Lastly, participants will be encouraged to continue LLIN use and not instructed to avoid alternative vector control tools (e.g., coils, topicals, aerosol sprays, repellents) which will allow for an estimation of the ET effect assuming all other measures are still occurring for malaria prevention, essentially providing insight on an additive benefit above that provided by currently recommended WHO malaria preventive measures.

#### Provisions for post-trial care {30}

Not applicable—the study will not provide post-trial care.

### Outcomes {12}

The primary outcome measure is the incidence rate of malaria infection as measured by active infection and clinical malaria case detection in cohorts of 55 children (between 6 months and 10 years old) per cluster, 17 clusters per arm on a biweekly basis in peak transmission season and monthly basis in low transmission season. [Time Frame: 24 months].

Secondary outcome measures include:

1. Clinical malaria incidence measured in children between 6 months and 10 years old living in the study cohorts using passive case detection via the existing community health workers and health centers. [Time Frame: 24 months]
2. Malaria parasitemia measured in children between 6 months and 10 years old in the cohorts of 55 children. [Time Frame: 24 months]
3. Prevalence of moderate (defined as 7–9.9 g/dL hemoglobin) to severe anemia (< 7 g/dL hemoglobin) measured in children under 5 years of age in the cohorts of 55 children four times: at the start and end of the rainy season (April and November respectively) of Year 1 and Year 2. [Time Frame: 24 months]
4. Mean numbers of female malaria mosquitoes (*An. gambiae s.l., Anopheles funestus s.l.*) captured in study houses measured by CDC light traps in 20 clusters, 10 houses per cluster on a monthly basis. [Time Frame: 24 months]
5. Malaria parasite sporozoite rate assessed in 10% of all anophelines captured by CDC light trap. [Time Frame: 24 months]
6. Entomological inoculation rates measured in each study arm as the product of the anopheline vector density and sporozoite rate. [Time Frame: 24 months]

Other pre-specified outcome measures include:

7. Cost modeling will assess the cost-effectiveness of EaveTubes compared to the previously applied Screening + EaveTubes intervention, and compared to other vector control interventions such as long-lasting insecticide nets and indoor residual spray. [Time Frame: 24 months]
8. Assessments of willingness to participate and adoption of EaveTubes at the end of the study period through questionnaires and willing-to-pay surveys. [Time Frame: 24 months]

### Participant timeline {13}

The duration of the current study is 2 years and 9 months. The first 6 months are for set up and baseline. The cRCT proper runs for 2 years with an additional 3 months for primary data analysis.

The epidemiological monitoring will require 1 to 2 visits per month, depending on the time of year (1 visit per month from November until April, and 2 visits during the peak season from May until October). The epidemiological monitoring visits are expected to last approximately 30 min, with an additional 30 min twice a year for anemia and respiratory testing.

The entomological monitoring will require 3 nights (6 pm–8 am) per house each month. ET installations will require approximately 3 months for an estimated 3000 households in 17 village clusters. Retreatment of netting inserts takes approx. 10 min per house and is done from the outside of the house.

**Table:** 

Study period				
	**Pre-trial**	**Baseline**	**Follow up**	**End of trial**
**Timepoint**	T_(Jan 2023–Feb 2023)_	T_(Mar 2023–Jun 2023)_	T_(Jul 2023–Jun 2025)_	T_(Jul 2025–Sept 2025)_
**Ramp-up**				
Village selection	X			
Community engagement	X			
Census	X			
Village randomization and allocation	X			
**Enrollment**				
Informed Consent		X		
Screening		X		
ET installation		X		
Distribution of LLINs		X		
Baseline prevalence study		X		
Parasite clearance		X		
**Intervention**				
Epidemiological monitoring (active case detection)			X	
Entomological monitoring			X	
Intervention monitoring and replacements			X	
**Assessments**				
Baseline Analysis		X		
Final Analysis				X

### Sample size {14}

The number of villages for the cRCT is estimated at 17 per treatment arm based on the power analysis which is detailed in the Statistical Analysis Plan.

The sample size determination on the required number of households per cluster for testing the primary hypothesis on PE is based on the hazard rate comparison in the proportional hazards regression model. With the following specifications: power = 80%, 2-sided type-I error rate = 5%.

- True PE/Minimum effect size = 35%
- Baseline first-time malaria infection hazard rate = 1.5 cases of falciparum malaria per person-year (conservative estimate based on control arm data from first SET cRCT)
- Coefficient of variation (k) = 40% (based on first SET cRCT)
- Loss to follow-up (LTFU) rate = 20%
- Monitoring = 2 years to capture 2 peak transmission seasons
- One interim analysis for efficacy and non-binding futility with the O’Brien-Fleming error spending function when 50% information is collected

With 17 clusters per treatment, 55 children (aged 0.5 to 10 years old) from 55 different households per cluster are expected to yield 1315 independent first-time malaria events with a 24-month follow-up period to yield 80% power in testing the primary hypothesis on PE. Since the sample size already factors in a 20% LTFU rate, there is no need for replacement subjects.

### Recruitment {15}

Local workers will be recruited from the trial village to enhance community engagement and study participation. Community sensitization procedures will include meetings with village leaders and inhabitants to introduce the study and each of its components.

#### Assignment of interventions: allocation

##### Sequence generation {16a}

The unit of randomization for the intervention and control will be a village cluster. For baseline, recruitment of participants for enrollment will be based on random HH selection following census and mapping of the study area. The study statistician will analyze baseline data to inform potential stratification criteria, i.e., baseline malaria incidence levels and/or adult entomological endpoints. Following stratification (as needed), allocation of individual villages to trial arms will be conducted using randomization. One of the eligible randomization allocations will be selected at a public ceremony in the presence of community leaders.

##### Concealment mechanism {16b}

Cluster allocation was randomly conducted using a lottery mechanism whereby pieces of paper will be marked ET and Control, placed in a basket, mixed, and then a randomly selected child will be asked to draw from the marked papers. Each drawn paper must correspond to a given village, until they are all listed out. This event will be a public ceremony in the presence of community leaders and the Ministry of Health.

##### Implementation {16c}

The study statistician will use a random number generator to generate the allocation sequence and assign clusters to treatment or control arms. Trained study staff will enroll participants to study clusters.

### Assignment of interventions: blinding

#### Who will be blinded {17a}

Given the nature of the intervention, it is impossible to conduct this study in a fully blinded manner but those parts of the data collection that can be blinded will be. Observer bias will be reduced where feasible. All laboratory work will be blinded. Mosquito collector bias will be reduced by using standard CDC light traps which do not rely on the ability of the fieldworker to collect specimens. Trap catches will not be examined and analyzed by those who collected them but by different technicians who will not know the trap location. We will use codes to identify any clinical samples. Electronic records will not carry the name of the research participants, only an alphanumeric code. Primary analysis by the project statistician will be conducted on blinded data (e.g., arms designated as treatment A and B or something similar).

#### Procedure for unblinding if needed {17b}

Datasets will only be unblinded once they have been locked.

### Data collection and management

#### Plans for assessment and collection of outcomes {18a}

##### Pre-trial

The pre-trial “ramp up phase” will be used to recruit and train local staff, and obtain ethical approval for the trial. During ramp up, forty candidate villages will be identified based on size (100–300 households) and proximity to Bouaké, Côte d’Ivoire, i.e., within a 50-km radius of Béoumi town in the Béoumi district of the Gbêkê region. We will select 34 of the 40 candidate villages based on ET suitability (no/small wall gaps, solid roof, no/small open eaves) and indications of participatory willingness based on meetings with the community leaders.

In each of the 34 trial villages, project personnel will meet with the authorities in each community (“chef du village”) to obtain permission to talk to the village residents about the trial. Village outreach will begin with town hall meetings to explain the trial and the possibility of a village being assigned to the treatment or the control arm, to outline all trial activities that will take place in the villages, and to answer any questions from the audience. A few days after the town hall meeting, the chef du village will be contacted to find out whether the community agrees to participate in the trial.

During ramp-up, a Population Census will be conducted in all the villages. Householders will be asked for information about their households (#, age, gender of family members in their HH) and given a new bed net if they have fewer than 2 per HH so that universal coverage is guaranteed in both trial arms. HH owners will be asked a short series of questions regarding their use of vector control tools, the availability of health care in the village, and markers of socio-economic status (house quality and availability of goods). For each HH, we will record the number of structures/dwellings, their construction (wall type, roof type, open eaves, wall gaps), number of windows, and number of doors. Following the local customs and local health ministry procedures in Côte d’Ivoire, each household (which typically does not have addresses) will be assigned a letter-number code that will be written in chalk on the door-jam of the house. During these activities, every eligible child in the village (between the ages of 6 months and 8 years old) will be assigned a unique identifier and their parents given a card with that unique identifier.

Community health workers will receive refresher training from a clinician before the onset of the trial baseline period.

##### Baseline (includes intervention installation)

Based on the Population Census data, 55 children in each village will be randomly selected for active monitoring of malaria infection. Informed consent will be obtained, and these children will be given ID cards for presentation to study nurses and health workers.

The baseline prevalence of malaria infection in the cohorts will be measured by taking blood samples from all children and confirming parasite infection using rapid diagnostic tests (RDTs). At the end of the baseline period, at the onset of the clinical phase, the entire study cohort will be cleared of malaria parasites, regardless of infection status, using a standard dose of first-line antimalarials.

Baseline entomological sampling will be done in all clusters in both arms, in 10 randomly selected HH per cluster. Once informed consent is obtained indoor mosquito collections will be done using CDC light trap collections during 3 consecutive nights per house. Subsets of 10% of baseline collected anophelines will be identified at species level and tested for sporozoites to quantify the baseline EIR in each trial arm.

Once census and baseline prevalence data have been obtained, randomization will be done to allocate the selected villages to the trial arms which will potentially be stratified by epidemiological, environmental, and/or demographic factors if it is necessary. At the end of the randomization process, there will be 17 villages in the control arm and 17 in the treatment arm. In the 17 treatment arm villages, heads of households that are suitable for ET installation will be offered the option of having ETs installed.

Following randomization, trial personnel will go door-to-door in the 17 selected intervention villages to obtain consent from the owners of eligible (ET suitable) houses for the installation of ETs. ET installation will start in parallel: commencing in village clusters as soon as > 50% consent in that particular village has been obtained. It is estimated that 3-month installation time is needed for the 17 ET clusters. There is an expectation of > 70% of HHs with ET installation in the intervention clusters, with 8–10 ETs per house on average. Standard Operational Manuals (developed from the first SET cRCT) will be used. It is estimated that 4-month installation time is needed for the 17 ET clusters.

##### Follow-up

At the start of the clinical follow-up period, the clean ET netting inserts will be replaced with the deltamethrin-treated ET inserts in all treatment clusters. This is estimated to take 2–3 weeks’ time.

Clinical follow-up will run for 2 years to cover two high transmission seasons (typically associated with the rains, May–October) and the remaining lower transmission periods.

**Epidemiological monitoring**

The incidence of malaria infection and clinical malaria will be determined by active case detection in febrile children in the study cohorts. Clinical monitoring and treatment will be performed by trained nurses from the Institut Pierre Richet, who will collaborate with the community health workers present in the trial villages. On the first visit, in the trial baseline period, parasite prevalence will be measured in all cohort children by RDT (SD Bioline Malaria Ag P.f/Pan; Standard Diagnostics; Seoul, South Korea). Immediately following this initial blood sampling, every child will be treated with a 3-day course of standard, first-line antimalarials (Coartem® or ASAQ Winthrop®, both ACTs recommended by the NMCP in Côte d’Ivoire) to clear any malaria parasite infections.

Children enrolled in the active detection cohort will be visited every 2 weeks during the peak malaria transmission season (May–October) and monthly during the rest of the year when transmission rates are low (November–April). At each visit, the clinical team will record the axillary temperature of each child. If the child is febrile or has a history of fever in the past 48 h or the parents report that their child was sick, the child receives a physical examination and a record will be made of symptoms. A finger prick blood sample will be taken from all febrile children. RDTs will be used to detect *Plasmodium* infection.

Children who are RDT-positive and are diagnosed with uncomplicated malaria by the study nurse will be treated immediately with first-line antimalarials for 3 days. This is the standard procedure for diagnosing and treating malaria in Côte d’Ivoire. Because it is a malaria-endemic region, only symptomatic children are typically treated. ACT treatment for malaria will be provided free of charge through the NMCP system. The community health worker who normally provides diagnostic and treatment in the village will be responsible for monitoring the child until he or she is cured. If the child exhibits any symptoms of severe illness, he or she will be sent immediately to the closest health clinic for treatment. Children who have been treated for malaria will be considered not at risk for 2 weeks following treatment and there will be no data collection for these individuals during this time. Nurses and community health workers will capture history of travel away from the household to flag any cases that might be a result of infection while traveling outside the village.

Clinical malaria incidence will also be monitored continuously by passive case detection using the existing clinical system: Community Health Workers and local health facilities. Any enrolled child between 6 months and 10 years old will be counted whenever they are brought to a community health worker or local health clinic with a body temperature of ≥ 37.5 °C. These data are planned to be provided weekly to ET Trial personnel.

Twice a year (at the beginning and end of the peak transmission season), all cohort children of 5 years of age or younger will have a blood sample taken for immediate measurement of anemia. Blood samples will be checked for hemoglobin levels using a spectrophotometer (Hemocue Hb diagnostic system).

**Entomological evaluations**

Deltamethrin will be the AI deployed in ET. In addition, the standard pyrethroid-only net distributed in the study area is PermaNet 2.0, which contains deltamethrin. Long-term efficacy of the insecticide-treated ETs will be evaluated using periodic WHO cone bioassays to validate continued impact against resistant wild-type mosquitoes.

We will assess the impact of ETs on entomological measures of malaria transmission by measuring the density of *Anopheles* mosquitoes indoors on a monthly basis: in 20 clusters (10 ET, 10 control) in 10 randomly selected households per cluster. Per trial arm, 10 new clusters will be selected each month so that all study villages will be sampled bimonthly, as recommended by the WHO VCAG. In the 10 selected treatment clusters, we will select only ET-treated households. Mosquitoes will be captured indoors overnight using miniature CDC light traps placed next to a sleeper under an LLIN for 3 consecutive nights. These traps will be placed inside the house by Vector Control Product Evaluation Centre/Institut Pierre Richet (VCPEC-IPR) entomology technicians at 6 pm in the evening and collected by the technicians at 8 am the following morning. Mosquitoes will be sorted by Household ID and date of collection. Indoor temperature and relative humidity will be recorded in enrolled HHs during entomological sampling using data logging devices.

A subset of 10% of all caught mosquitoes will be identified by microscopy and the numbers of *An. gambiae* s.l. and other species recorded. In subsets of 10% of all caught anophelines, we will type to species level using PCR. Results will be used to estimate mean vector density and species composition per trial arm. For the *An. gambiae* and *An. funestus* samples in these subsets, we will assess the presence of sporozoites using CSP-ELISA. Sporozoite prevalence will be measured from a random sample of up to 60 anopheline females per cluster per sampling night.

We will estimate the EIR in each study arm (i*.*e.*,* mean number of sporozoite infective per bites per cluster per month) where we assume that a mosquito caught indoors and actively searching is able to bite the host.

$$\mathrm{EIR}=\mathrm{Human}\;\mathrm{biting}\;\mathrm{rate}\;\times\;\mathrm{Sporozoite}\;\mathrm{rate}\;\times\;365=\frac{\mathrm{Number}\;\mathrm{of}\;\mathrm{mosquitoes}\;\mathrm{collected}}{\begin{array}{c}\mathrm{Number}\;\mathrm{of}\;\mathrm{capture}\;\mathrm{nights}\\\end{array}}\times\frac{\mathrm{Number}\;\mathrm{sporozoite}\;\mathrm{positive}\;\mathrm{mosquitoes}}{\begin{array}{c}\mathrm{Number}\;\mathrm{of}\;\mathrm{mosquitoes}\;\mathrm{tested}\\\end{array}}\times365$$

**Intervention quality monitoring**

Integrity of the intervention (e.g., damage to the ET inserts) in the treatment villages and general house condition in all villages will be monitored by quarterly village walk-throughs every 4 months by project staff in both study arms. In addition, a designated member of the study team will be available for householders to report ET-related construction problems and get them fixed during the trial.

The persistence of the chemical insecticide used on the inserts will be monitored bimonthly by taking a sample of inserts from the treated villages to the lab (these will be replaced with fresh inserts) and exposing mosquitoes to them in a controlled bioassay. We will use F1 adult female anopheline mosquitoes reared from field-collected eggs in WHO cone tests with a 3-min exposure and mortality monitored 1 day post-exposure. Mortality will be compared against equivalent mosquitoes (wild-type resistant) exposed to untreated “control” inserts. Inserts will be replaced once the mortality post-exposure falls below 70% (from previous results, we expect this to be every 10–12 months).

**Data forms**

A combination of standardized paper-based or digital forms (under Android tablets) will be used. VCPEC-IPR and University of Notre Dame (UND)/Center for Research Computing (CRC) will work together to develop the quantitative forms to be uploaded on Android tablets. Entered data (entomological and epidemiological) will be automatically assessed for quality using established quality control rules, then reviewed, and appended to the data already present. Data forms can be made available upon request submitted to UND.

##### End of trial

The final clinical survey of the child cohort will take place at the end of the 24-month follow-up. The final light trap collection will take place at about the same time.

At the end of the trial, HH owners will be offered to have the ETs blocked with a closed insert or plastic cap to block the tubes. However, we intend to liaise with stakeholders throughout the trial to gage interest in providing continued retreatment for ET inserts after the trial, as is now taking place in Côte d’Ivoire.

##### Sub-studies

The sub-studies described in this section are not part of the WHO-approved clinical study design as they are secondary endpoints and not part of the primary evaluation of public health impact of the intervention.

We will conduct an assessment of key user acceptance indicators and cost-effectiveness of the ETs intervention vis-a-vis other alternative technologies or practices available. These sub-studies will be critical for successful scaling of the product, as they will help identify the most socially acceptable and sustainable way of achieving and maintaining high coverage of ETs. User acceptance does not always translate into adoption unless the intervention is found to be affordable and more cost-effective compared to other available options for malaria protection. In this regard, cost-effectiveness analyses are planned in this study.

**Cost-effectiveness studies**

A Global Health Economist within UND’s Keough School of Global Affairs will conduct an economic evaluation to estimate the costs and cost-effectiveness of the ETs intervention. The cost analysis will take the gold-standard, societal perspective which includes both provider and community costs. Implementation costs will be carefully monitored during ETs installation and maintenance activities. Data on incremental costs of the ETs product, house modifications during install, and their sources will be collected from project expenditure records. Household costs will be collected at cohort enrolment shortly after intervention installation, and the unit costs (cost per house and person) will be calculated. This cost study will demonstrate the costs of using ETs as a stand-alone tool compared to the previously combined intervention of SET.

The cost-effectiveness analysis will have the primary endpoint of cost per disability-adjusted life years (DALYs) averted by ETs. Incremental cost per DALY averted for ETs (Intervention arm) relative to LLINs alone (control arm) will be calculated using the epidemiological data collected during the trial. Cost-effectiveness ratios will be presented as point estimates and ranges (using the confidence intervals (CIs) on the epidemiological data and reflecting any uncertainty in costs) and interpreted against a range of willingness to pay thresholds and in relation to the cost-effectiveness of other malaria control interventions.

Cost modeling will assess the cost-effectiveness of ETs compared to the previously applied SET intervention, and compared to standard vector control interventions such as LLINs and IRS. The potential cost-effectiveness of ETs at scale over time will be simulated to facilitate comparison with other malaria control interventions and cost-effectiveness benchmarks. We will measure the cost of ETs’ implementation in relation to manufacturing, efficacy, and coverage to model projections of cost-effectiveness to incentivize potential procurers.

**User acceptance studies**

Social scientists from the Institut Pierre Richet will use quantitative methods to assess ETs’ adoption, adherence, and acceptability among study participants. Combined, endpoints from these assessments will inform potential bottlenecks to product access, uptake, and implementation post-trial.

Ethnographic data will be collected during the baseline population census to inform socio-economic status of households and participants. Adoption will be assessed using indicators of informed consent rates and ETs coverage rates in the trial. Adherence will be assessed via quality control monitoring of ETs’ integrity during the trial. User acceptance will be measured based on inhabitants’ willingness to pay and continue using ETs after the trial through an endline survey.

To design potential strategies for scale-up, we will identify and engage with key local, national, and international level stakeholders, involving them in discussions and creating a driving team to identify mechanisms for expansion and institutionalization while acting as advocates for the innovation.

#### Plans to promote participant retention and complete follow-up {18b}

Participant retention strategies include fostering relationships with participants, provision of community health worker and nurse contact information for easy communication to alleviate concerns, and periodic generation of retention rates to evaluate strategies.

#### Data management {19}

UND will achieve a systems approach to protocol adherence, implementation, data gathering, and sharing by installing a program monitoring plan (i.e., data gathering, training oversight, overall program progress) that will support rigorous data collection for policy recommendation. The UND CCRC will host the data server and will be responsible for data management, data form processing, and provision of support for training in-country collaborators’ data entry personnel.

Hardcopies of study and study-related documents (e.g., protocols, raw data, documentation, and final reports generated during a study, as well as chemical usage sheets) will be stored at VCPEC-IPR in a locked filing cabinet under the supervision of a designated archivist. Electronic data will be stored on UND servers with restricted access.

After study files have been held in the VCPEC-IPR for 5 years, the archivist will contact the sponsor to determine their future storage requirements. At the request of the Sponsor, it will either continue to be kept at VCPEC-IPR, sent to the Sponsor for storage, or destroyed. All hardcopies of documents will be destroyed by shredding and data on disks will be wiped, and the disks broken prior to disposal in the VCPEC-IPR waste.

#### Confidentiality {27}

Study subjects will be identified only by their study identification number and any electronic database will only contain their study identification number. Personal identifiers will be removed from the transcripts of interviews and discussions with participants being identified only through a study identification number. Thus, all data will be anonymized on data entry. The UND CRC databases will be password-protected and accessible only to authorized personnel. All hardcopy documents will be securely stored in locked filing cabinets and accessible only to authorized personnel.

The PI will maintain appropriate medical and research records for this study in compliance with the principles of good clinical practice and regulatory and institutional requirements for the protection of confidentiality of participants.

The authorized representatives of the sponsor, the ethics committee(s), or regulatory bodies may inspect all documents and records required to be maintained by the investigator, including but not limited to, medical records (office, clinic, or hospital) for the participants in this study. The clinical study site will permit access to such records.

#### Plans for collection, laboratory evaluation, and storage of biological specimens for genetic or molecular analysis in this trial/future use {33}

Not applicable. Study has no planned future uses for the biological specimens.

### Statistical methods

#### Statistical methods for primary and secondary outcomes {20a}

The baseline characteristics of the enrolled subjects, households, and clusters will be summarized by treatment arm (control arm, LLIN and intervention arm, ET + LLIN). Specifically, we will examine subject age and gender at the individual level, wall type and roof type, house open eaves, number of windows, number of doors at the household levels, and cluster population and baseline prevalence rate at the cluster level.

In the following analysis that involves malaria incidence, a child will not be considered at risk for malaria for 2 weeks after any treatment for malaria.

##### Primary

Protective efficacy (PE) against clinical malaria infection will be determined by comparing hazard rates of malaria clinical cases between the two treatment arms based on an intention to treat (ITT) analysis. The primary hypothesis on PE against overall malaria case incidence will be tested by comparing the hazard rates of the overall malaria case incidence between the control and ET in the ITT population using a proportional hazards model. The model will include relevant individual-level, household-level, and cluster-level baseline covariates, treatment assignment, and follow-up visits and random effects to account for correlations among the subjects within the same cluster and among multiple infections within the same individual. The hazard ratio $\beta$ between ET and control will be estimated, along with a 95% confidence interval. The null hypothesis of PE = 0% is equivalent to $\beta =0$, which will be tested by the Wald’s test $z=\widehat{\beta }/s$, where $s$ is the estimated standard error of $\widehat{\beta }$. There will be one formal interim analysis to test the primary hypothesis. The decision boundaries are calculated for either stopping for futility or stop for efficacy using the O’Brien-Fleming error spending function [8–10], with a 2-sided Type I error rate of 0.05, Type-II error rate of 0.2; baseline incidence rate of 1.5 per person-year, and between-cluster CV of 40%. The interim analysis will occur when 658 events (50% information) are collected. Assuming a baseline incidence rate of 1.5 per person-year, the interim analysis is estimated to occur around the end of Year 1 of the intervention follow-up. Due to the formal interim look at the data which costs a certain amount of type-I error rate and type-II error rates, the final critical value is different from what would be for a study without the interim look.

If the z-score of the log(hazard ratio) from the comparison between ETs and control at the interim look is >  − 0.7288 then the study can stop for futility (if *z*-score > 2.7965, the trial would stop for definitive harmful effects of ETs though the chance this would occur is close to 0 if not 0), the study can stop for futility. Since we adopt the non-binding futility boundary the study can continue to collect more data even if we cross the futility boundary at the interim look, there will be no inflation of type I error. In other words, the trial does not need to stop to accept the null hypothesis when the test statistic falls in the futility region at the interim stage. If the interim z-score <  − 2.7965 then the study can stop for efficacy. The study may also continue even if the efficacy boundary is crossed at the interim look, and there will be no inflation of type I error as efficacy is already established at the interim. If the interim z-score falls within (− 2.7965, − 0.7288), the study continues.

If the interim results do not cross either the futility or efficacy boundaries, the *z*-score from the final analysis upon the completion of the study will be compared with − 1.9744. If the *z*-score <  − 1.9744, we reject the null hypothesis, claiming ETs reduce the malaria hazard rate compared to control at the significance level of 5%; if the *z*-score > 1.9744, we fail to reject the null hypothesis, claiming ETs do not reduce the malaria hazard rate compared to control in Cote d’Ivoire. If the interim results cross either the futility or efficacy boundaries, the final analysis is only conducted for estimation purposes, as the futility or efficacy of ETs is already established at the interim.

##### Secondary

**PE of ETs against malaria infections (both with clinical symptoms and asymptomatic)**

A similar proportional hazard model used for analyzing the primary endpoint above will be applied. The malaria hazard ratio between ET and control will be estimated, along with 95% CI.

**PE of ETs against malaria infections (both with clinical symptoms and asymptomatic)**

A similar proportional hazard model used for analyzing the primary endpoint above will be applied. The malaria hazard ratio between ET and control will be estimated, along with 95% CI.

**PE analysis without baseline covariates**

A PE analysis of clinical malaria infection will be also performed by removing all the baseline covariates from the proportional hazards presented above and keeping the treatment arm as the only covariate in the proportional hazard model. The hazard ratio between ET and control will be provided, along with a 2-sided 95% CIs. A similar analysis will be performed for clinical and asymptomatic malaria infections combined.

**Anemia**

The first study on ET [14] suggests ET has a protective effect against anemia. In this study, an in-depth health examination of the recruited children for malaria follow-up will be conducted at the beginning and the end of the transmission season in both Year 1 and Year 2 to monitor severe anemia. A mixed-effects logistic regression will be used to compare ETs and control in the anemia proportion. The model has anemia status (Y or N) as the outcome; a similar set of covariates as in the proportional hazards model in the primary endpoint analysis is used, with two additional terms of time (baseline, Year 1, Year 2) and time-by-treatment interaction, along with random effects to count for correlations among the subjects within the same cluster and among multiple anemia episode within the same subject, used. The regression coefficient associated with treatment arm quantifies the ratio between the ET and control arms on the odds of getting anemia, which will be estimated, along with a 95% confidence interval.

**Effect of ETs on malaria prevalence**

Malaria prevalence data will be collected at baseline and at the end of Year 1 and Year 2. A mixed-effects logistic regression as used in the anemia analysis above will be used to compare ETs and control on malaria prevalence over time, with the outcome anemia status being replaced malaria status (Y or N).

**Incidence rate**

The malaria incidence rate is defined as the ratio of the number of new malaria cases during the follow-up period vs the sum of the time at risk (in year) across the individuals within the same cluster. The incidence rates per person-year during the whole intervention follow-up will be calculated by cluster in the ET and the control arms respectively. Summary statistics of the cluster-level incidence rate will be provided by treatment arm; the incidence ratio between the two arms will be also calculated.

**Effects of ETs on entomology**

The endpoints in the entomological analysis include anopheline density collected by light-trap, anopheline sporozoite rate, and anopheline EIR.

We will report the frequency and proportion of each anopheline mosquito Genus and species collected using a light trap for each cluster and by treatment arm. The time profile plots of overall anopheline density will be obtained at the baseline and during the intervention period. An appropriate statistical model for anopheline density will be identified after examining the distributional characteristics of the density data, which are likely to follow (zero-inflated) Poisson distribution or (zero-inflated) negative binomial distribution if there is over-dispersion. The covariates in the models will include fixed effects of treatment, time, cluster population size, number of houses in a cluster, and a random effect for cluster. The ratio between ET and control in anopheline density will be estimated along with a 95% CI; the %reduction in anopheline density by ET is given by (1 − density ratio) × 100%.

The analysis for sporozoite rate will be similar to that for analyzing mosquito density If the data on sporozoite positivity are highly unbalanced in the sense that the marginal distribution of the variable (e.g., 99% negative sporozoite), then the model might lead to unstable estimates or the model might not even converge. The ratio between ET and control in sporozoite rate will be estimated along with a 95% CI; the %reduction in sporozoite rate by ET is given by (1 − sporozoite rate ratio) × 100%. In such cases, only summary statistics will be provided. EIR is the product of the anopheline vector density and the sporozoite rate and its analysis is similar to what’s used for analyzing sporozoite rate. Summary statistics will be provided on sporozoite rate and EIR at baseline and per year during the intervention period by treatment group.

**Analysis of the relationship between malaria hazard rate and entomological endpoints**

To explore the relationship between the malaria hazard rate and the entomological endpoints, a similar model as the proportional hazards regression model used to address the primary objective on the malaria infection will be applied to the clusters from which the entomological data are collected, with similar random effects specification. For the covariates in the model, in addition to those used for analyzing the primary endpoint, we will also include a covariate that captures the entomological information; whether it is a linear or non-linear term will be informed by the exploratory data analysis of the relationship between the cluster-level incidence rate and each of the entomological endpoints (e.g., indoor mosquito density and sporozoite positivity rate if there is enough data). The regression coefficient associated with the entomological covariate quantifies how the entomological covariate affects the malaria hazard rate.

**Safety assessment**

Mean, minimum and maximum frequency, and percentage of AEs and SAEs across clusters among enrolled subjects will be summarized by treatment arm. The AE/SAEs summary will be provided for both clinical diagnosis and symptoms. In addition, they will be labeled as Probable, Possible, Plausible (such as dermal events, oral events, Inhalation events), or Unlikely (eye Irritation, headache) due to ETs.

#### Interim analyses {21b}

A formal interim analysis during the intervention period will be conducted to test the primary hypothesis as outlined above. The interim analysis will be performed by an independent statistician on the DSMB, and the interim outcome report will be shared only with In2Care and VCAG, not with UND or Cote d’Ivoire investigators to mitigate unintentional bias in data collection in the remaining period of the study after the interim analysis. The final decision to stop should always rest with the DSMC, not the investigators, or the funder.

#### Methods for additional analyses (e.g., subgroup analyses) {20b}

The analysis outlined in the primary and secondary analysis will be based on the intent-to-treat (ITT) dataset. The study will also examine the per-protocol (PP) dataset. If the PP dataset differs significantly from the ITT dataset for a particular analysis, the analysis will also be performed in the PP dataset.

#### Methods in analysis to handle protocol non-adherence and any statistical methods to handle missing data {20c}

Significant effort will be made to avoid having missing values on outcome (malaria infection status and visit dates, and entomological endpoints). When missing values occur for an outcome for reasons not related to the outcome, reasons for missingness and the missing fraction by treatment arm and cluster will be reported. Per protocol, the subjects are screened actively on the malaria status (the outcome) every 4 weeks.

If a subject misses one or more scheduled visits due to reasons not related to the ETs product or the outcome, the subject will have missing values on the outcome that can be regarded as ignorable missingness (MAR or MCAR [11]).

If a subject drops out study due to reasons unrelated to the ETs product and/or malaria infection, then the missing observations from the subject can be regarded as ignorable missingness (MAR or MCAR [11]).

In both cases, all the available data from the subject will be included in the primary and secondary analysis, without employing any specific missing data analysis techniques, due to the ignorability of the missing mechanisms [11].

Missing baseline covariates (individual-level, household-level, and cluster-level) that are a part of the regression models for the outcome of interest will be imputed using simple hot-deck imputation methods if the missing fraction for the covariate is < 5%. If the missing fraction for a covariable is ≥ 5%, appropriate multiple imputation approaches will be applied. If ≥ 50% of the subjects have missing values on a covariate (due to missing at random or missing completely at random), that covariate will be excluded in the model.

#### Plans to give access to the full protocol, participant-level data, and statistical code {31c}

The SAP and analytic code will be made open access. Data and supporting information will be made available 12 months following the completion of data analysis and will remain open access in the public domain.

### Oversight and monitoring

#### Composition of the coordinating center and trial steering committee {5d}

In2Care will serve as the lead organization for this program and will assume the overall responsibility for management, oversight, and administration for the program. The coordinating personnel at UND will include the Lead PI, Co-Investigators, Program Manager, and Finance Manager. UND will communicate on a day-to-day basis with In2Care and VCPEC-IPR. VCPEC-IPR will be responsible for running the cRCT on a day-to-day basis which includes but will not be limited to conducting a baseline survey, deploying ETs, entomological monitoring, and subject follow-up. Representatives from VCPEC-IPR and UND will all serve on the data management team to oversee the development and implementation of data collection, recording, and cleaning.

A Trial Steering Committee (TSC) will be established. Members on the committee will have the clinical, epidemiological, and statistical expertise to monitor study progress and safety of participants, and the committee will have access to the study data. SOPs will be developed, adhering to Good Clinical Practice and Good Field Entomology Practice.

#### Composition of the data monitoring committee, its role, and reporting structure {21a}

The trial will not consist of a data monitoring committee as the TSC will serve in this capacity. In addition, routine data monitoring and management for Quality Assurance and Quality Control will be conducted in partnership between UND and VCPEC-IPR data management teams.

#### Adverse event reporting and harms {22}

Safety oversight will be carried out by the TSC. Children of the study cohorts will have access to the current standard of care. Serious adverse events (SAEs), whether attributed or not to ETs or LLINs, will be recorded throughout the trial. The host institution medical expert (Dr. Serge Assi) and his clinical team will be responsible for recording, reporting, and managing SAEs, including follow-up, in accordance with national guidelines. A summary table of all SAE will be provided at regular intervals to the TSC.

#### Frequency and plans for auditing trial conduct {23}

The Chief Investigator will permit study-related monitoring, audits, and inspections by the study sponsor, IRB, TSC, and government regulatory bodies, of all study-related documents (e.g., source documents, regulatory documents, data collection instruments, study data, etc.). The investigator will ensure the capability for inspections of applicable study-related facilities (e.g., pharmacy, diagnostic laboratory, etc.).

#### Plans for communicating important protocol amendments to relevant parties (e.g., trial participants, ethical committees) {25}

All amendments to the protocol will be documented. Substantial amendments will require a report and approval by the TSC and/or the ethical review boards.

#### Dissemination plans {31a}

Dissemination of results includes submission to WHO VCAG, workshop with study partners, on-site meetings in Cote de Ivoire, and presentations at scientific meetings and/or peer-reviewed publications.

## Discussion

Control efforts in sub-Saharan Africa over the past 15 years have prevented millions of clinical cases of malaria caused by *Plasmodium falciparum* [1]. Vector control tools, LLINs and IRS, are estimated to be responsible for a majority of those averted cases [2]; however, insecticide resistance has seriously hampered their efficacy in recent years. Because of the impact of vector control on malaria transmission, new tools which are cost-effective, environmentally friendly, and socially acceptable need to be developed to complement the existing arsenal.

In2Care® ETs could help meet the pressing need for new vector control tools. ETs are an inexpensive, field-ready technology, informed by mosquito ecology to efficiently target and kill malaria vectors. In a previous cRCT conducted in Côte d’Ivoire, ETs + window screenings were shown to reduce malaria case incidence by 38% and by 47% in villages with > 70% coverage [14]. The objective of the current trial is to further demonstrate the epidemiological and entomological impact of ETs and generate additional data required by WHO to assess public health value.

## Trial status

Protocol version 4.0 from July 17, 2022. The study has currently completed the baseline prevalence survey (as of June 22, 2023), whereby participants were recruited, screened, and enrolled for confirming parasite infections using RDTs. Baseline data analyses are ongoing to verify underlying assumptions of malaria prevalence and coefficient of variation. Recruitment, screening, and enrolment of subjects for follow-up with intervention are scheduled to commence in August 2023.

### Supplementary Information

**Additional file 1.**

## Data Availability

The statistical analysis plan and analytic code will be made open access. The data and supporting information will be made available 12 months following completion of data analysis and will remain open access in the public domain. Open-access repository distributed under the terms of the Creative Commons Attribution (CC-BY) License, which permits unrestricted use, distribution, and reproduction in any medium, provided the original author and source are credited.

## Abbreviations

ACT: Artemisinin combination therapies
AE: Adverse event
AI: Active ingredient
CRC: Center for Research Computing
cRCT: Cluster randomized control trial
CI: Confidence interval
DALY: Disability-adjusted life year
ET: EaveTube
EIR: Entomological Inoculation Rate
HH: Household
ID: Identification
IRS: Indoor residual spray
VCPEC-IPR: Vector Control Product Evaluation Centre/Institut Pierre Richet
ITT: Intention to treat
LLIN: Long-lasting insecticide net
LTFU: Loss to follow-up
MAR: Missing at random
MCAR: Missing completely at random
NMCP: National Malaria Control Program
INSP: National Institute of Public Health of Côte d'Ivoire
PE: Protective efficacy
PI: Principal investigator
RDT: Rapid diagnostic test
SAE: Serious adverse event
SAP: Statistical Analysis Plan
SET: Screening + EaveTubes
TSC: Trial Steering Committee
UND: University of Notre Dame
VCAG: Vector Control Advisory Group
WHO: World Health Organization
